# Leicester Cough Questionnaire validation and clinically important thresholds for change in refractory or unexplained chronic cough

**DOI:** 10.1177/17534666221099737

**Published:** 2022-05-25

**Authors:** Allison Martin Nguyen, Jonathan Schelfhout, David Muccino, Elizabeth D. Bacci, Carmen La Rosa, Margaret Vernon, Surinder S. Birring

**Affiliations:** Merck & Co., Inc., Rahway, NJ, USA; Merck & Co., Inc., Rahway, NJ, USA; Merck & Co., Inc., Rahway, NJ, USA; Evidera, Seattle, WA, USA; Merck & Co., Inc., Rahway, NJ, USA; Evidera, Bethesda, MD, USA; Centre for Human & Applied Physiological Sciences, School of Basic & Medical Biosciences, Faculty of Life Sciences & Medicine, King’s College London, Denmark Hill, London SE5 9RS, UK

**Keywords:** clinically meaningful change, cough-related quality of life, Leicester Cough Questionnaire, patient-reported outcomes

## Abstract

**Introduction:**

The Leicester Cough Questionnaire (LCQ), a cough-specific quality-of-life measure, evaluates the impact of cough across physical, psychological, and social domains in patients with chronic cough (CC). This study assessed the psychometric properties of the LCQ.

**Methods:**

Data from a phase IIb, randomized controlled trial of the P2X3-receptor antagonist gefapixant were analyzed (NCT02612610). Subjective [Cough Severity Diary, cough severity visual analogue scale, and patient global impression of change (PGIC)] and objective (awake and 24-h cough frequency) data were used to validate the LCQ for use in patients with refractory or unexplained CC (RCC and UCC, respectively). Psychometric analyses included confirmatory factor analyses, internal consistency and test–retest reliability, validity, responsiveness, and estimated within-patient thresholds for clinically meaningful change.

**Results:**

Model-fit values for the proposed three-factor LCQ domains and most individual items were acceptable. Analyses suggest that a mean improvement ranging from 1.3 to 2.3 points for the LCQ total and ⩾0.8, ⩾0.9, and ⩾0.8 points for physical, psychological, and social domain scores, respectively, had the best sensitivity and/or specificity for predicting patient ratings of improvement on the PGIC.

**Conclusions:**

The LCQ is a valid and reliable measure to evaluate cough-specific quality of life and is a fit-for-purpose measure for use in patients with RCC or UCC. Although a single threshold for defining clinically meaningful change depends on the context of use, the results can help guide both treatment decisions and drug development. Therefore, clinicians may consider a ⩾1.3-point increase in the LCQ total score as clinically meaningful.

## Introduction

Chronic cough (CC; cough lasting >8 weeks) is a bothersome condition that can have a profound impact on health-related quality of life (HRQOL).^1–3^ Although CC may be associated with an underlying condition (e.g., asthma, upper-airway cough syndrome, or gastrointestinal reflux disease), some patients experience cough that persists despite optimal treatment of underlying conditions according to practice guidelines [refractory CC (RCC)] or have no identified diagnosable cause of cough despite systematic medical evaluation [unexplained CC (UCC)].^1,4^ Both RCC and UCC can persist for years.^3^ Patients with CC may experience cough-related physical, psychological, and social burdens that can impact their daily activities.^5,6^ This impact has been characterized by several distinct but interrelated components of cough severity, including cough frequency, cough intensity, disruption of daily activities due to cough, and cough-specific HRQOL.^7^ To obtain a comprehensive understanding of the impact of CC across these components, patient-reported outcome (PRO) measures are used. Guidelines for assessing outcomes in CC studies recommend using objective and subjective endpoints, with the latter assessed through the use of valid and reliable PRO measures.^3^

The Leicester Cough Questionnaire (LCQ) is a PRO tool that measures the impact of cough on patients’ lives and is among the most frequently used and studied cough-specific HRQOL instruments.^8,9^ The LCQ captures the impact of cough over the prior 2 weeks across physical, psychological, and social domains.^8^ Studies have shown that the LCQ is valid, reliable, and responsive in measuring the impact of CC in adults and adolescents.^8,10^ The LCQ has also been validated in acute cough, cystic fibrosis, bronchiectasis, and chronic obstructive pulmonary disease^9,11–13^ and has been translated into and validated for use in several languages.^14–20^

Although previous research established the minimally important difference as a 1.3-point change in the LCQ total score in patients with CC,^21^ this was a small (*N* = 52), observational study that used a limited number of analyses to determine minimally important difference. As few studies have assessed the minimally important difference of the LCQ, further estimates for meaningful LCQ change thresholds are needed. Therefore, this study used data from a phase IIb, randomized controlled trial for individuals with RCC or UCC to assess the psychometric properties of the LCQ, including test–retest reliability, convergent validity, known-groups validity, and responsiveness. This study also sought to estimate further thresholds for meaningful changes in the LCQ total and domain scores to help clinicians identify patients with RCC or UCC who may be considered responders to cough interventions.

## Methods

### Data source

This *post hoc* analysis used LCQ and other outcomes data collected in a phase IIb, 12-week study of the P2X3-receptor antagonist gefapixant (NCT02612610), for which results have been reported elsewhere.^22^ Key eligibility criteria included a diagnosis of RCC or UCC (as defined by American College of Chest Physicians and British Thoracic Society guidelines) for ⩾1 year, a cough severity visual analogue scale (VAS) score ⩾40 mm at screening, and no substantial abnormalities contributing to cough within the past 5 years (determined by chest X-ray). Participants were administered one of three doses of gefapixant or matching placebo. Blinded data from all randomized and treated participants (i.e., both placebo- and active-treated participants) were pooled into a single data set for all analyses reported in this article.

### Outcome measures

The LCQ is a 19-item cough-specific HRQOL questionnaire containing physical, psychological, and social domains (Supplementary Figure S1). Scores for the LCQ include mean scores for each domain (ranging from 1 to 7) and a total score calculated as the sum of the domain scores (ranging from 3 to 21). Each LCQ item assesses symptoms, or the impact of symptoms, on HRQOL over the past 2 weeks using a 7-point Likert-type scale ranging from *all of the time* to *none of the time*. Higher scores indicate better HRQOL.^8^ The LCQ was administered at baseline and weeks 4, 8, and 12.

This analysis used several other measures collected in the phase IIb trial to validate the LCQ, including the Cough Severity Diary (CSD), cough severity VAS, patient global impression of change (PGIC), and objective cough frequency. Each measure has been previously described;^23^ additional details pertaining to these measures can be found in the Supplementary Methods. Primary cough frequency analyses were conducted using awake cough frequency (the primary endpoint for the phase IIb study); additional analyses using 24-h cough frequency were also conducted and are reported in the Supplementary Materials.

### Statistical analyses

To minimize potential recall bias at later time points, we report primary analyses at week 4. Descriptive statistics are summarized at baseline and follow-up. Missing data are reported as the percentage of overall frequency, as well as the number of participants with more than one missing item. Observed data were used for all analyses with no data imputation. In the case of missing data, pair-wise deletion was employed.

#### Domains and confirmatory factor analyses

Methods for the domains and confirmatory factor analyses can be found in Supplementary Methods.

#### Internal consistency and test–retest reliability

Internal consistency was assessed using Cronbach’s α for the LCQ total score and each domain at baseline and weeks 4 and 8 to evaluate consistency over time. To assess test–retest reliability, intraclass correlation coefficients (ICCs) and score changes using paired *t* tests were calculated between the relevant initial LCQ scores (baseline) and retest LCQ scores (week 4) among a subset of participants categorized as *stable* during that time frame. The cough severity VAS (⩽10mm change) and awake cough frequency (⩽10% change) were the primary tools used to define a *stable* population; PGIC (report of *no change*) was used as a secondary metric. Reliability was interpreted as poor (ICC <0.05), moderate (ICC 0.5 to <0.75), good (ICC 0.75 to <0.90), and excellent (ICC >0.9 when a true stable population has been defined).^24^

#### Validity and responsiveness

Convergent validity was evaluated as the magnitude of correlations between the LCQ total and domain scores and other similar constructs (i.e., CSD and VAS) at week 4. Correlation coefficients were interpreted as low (absolute magnitude of 0.30 to <0.50) and moderate (⩾0.50 to 0.70).^25^

Known-groups validity refers to the degree to which a measure is able to discriminate between levels of disease severity. To assess the known-groups validity of the LCQ, three levels of disease severity were defined based on tertiles of awake cough frequency; the sample distribution of awake cough frequency at baseline and again at week 4 was used to stratify participants into tertiles of disease severity. Analysis of variance was used, with *post hoc* category comparisons via the Scheffé test to determine whether LCQ scores discriminate between the three awake cough frequency severity groups at each time point.^26^

To evaluate responsiveness, analysis of covariance was used to compare changes in LCQ total and domain scores from baseline to week 4 by response on the PGIC at week 4, controlling for baseline LCQ scores. This analysis was replicated using percentage change in awake and 24-h cough frequency to define responders and nonresponders using four definitions: (1) ⩾30% reduction, (2) ⩾50% reduction, (3) ⩾70% reduction, and (4) ⩾0.30 standard deviation (SD) reduction (or ⩾30% reduction in SD) using a distribution-based approach. In addition, as recommended by the US Food and Drug Administration,^27^ effect sizes were calculated for the LCQ total and domain scores for the awake cough frequency groups as defined above. Effect sizes were interpreted as small (0.20), moderate (0.50), or large (0.80).^28^

### Clinically meaningful change threshold

Anchor-based and distribution-based approaches were used to estimate clinically meaningful within-patient change thresholds for the LCQ total and domain scores. Distribution-based approaches included calculation of one-half of the SD of the LCQ total and domain scores at baseline and the standard error (SE) of measurement, estimated by multiplying the baseline SD of the LCQ by the square root of (1 − ICC).

In the anchor-based approach, the mean changes in LCQ scores from baseline to week 4 were calculated for patients in each PGIC category at week 4. The PGIC was selected as the anchoring questionnaire because it is widely used and has fewer response categories than other similar questionnaires (e.g., the Global Rating of Change Questionnaire), making it easier for patients to use. The anchor-based approach to estimate a meaningful within-patient change in the LCQ total score was supplemented with empirical cumulative distribution function (eCDF) and empirical probability density function (ePDF) curves. Specifically, the distribution of LCQ total score changes from baseline to week 4 was plotted for each response category of the PGIC to identify whether a range of LCQ total score changes could be considered meaningful.

Receiver operating characteristic (ROC) curves were evaluated to identify changes in LCQ total and domain scores from baseline to week 4 with the best sensitivity and specificity for predicting participants scoring a 1 to 3 (*very much improved, much improved, minimally improved*) *versus* 4 to 7 (*no change, minimally worse, much worse, very much worse*) on the PGIC. Changes in LCQ scores with the best sensitivity and specificity for predicting participants scoring a 1 to 2 *versus* 3 to 7 on the PGIC were also assessed. The Youden index (sensitivity + specificity − 100) was used to identify the point on the ROC curve where sensitivity and specificity of LCQ changes were optimized for predicting the PGIC outcome.

The results of the anchor- and distribution-based approaches were triangulated to estimate a range of thresholds for LCQ total and domain scores that could be considered meaningful within-patient changes.

## Results

### Participant population

Baseline characteristics of the study sample have been previously reported.^23^ Of the 253 participants enrolled, the majority were female (76.3%) and White (92.9%), with a mean (SD) age of 60.2 (9.9) years. Almost all participants (97.6%) had used medication to treat their cough within 30 days of screening.

### Missing data

Missing data were minimal because most measures (LCQ, cough severity VAS, PGIC, cough frequency) were completed during clinical visits and were reviewed by the site study coordinator for completeness. At baseline, no data were missing from the LCQ or cough severity VAS; CSD data (completed daily via electronic diary) were missing for 5.5% of participants. At week 4/week 8, data were missing from the LCQ (1.3%/0.9%), cough severity VAS (1.3%/0.4%), PGIC (1.7%/0.4%), and CSD (7.1%/7.0%).

### LCQ change

The mean (SD) LCQ total score improved at each time point over the course of the study, from 11.7 (3.0) at baseline to 14.7 (3.6) at week 4. The full range of LCQ item scores (i.e., from 1 to 7) was utilized for each LCQ item, and there were no floor or ceiling effects observed for the total or domain scores. Item-level descriptive statistics of the LCQ are provided in Supplementary Table S1.

### Domains and confirmatory factor analyses

Domains and confirmatory factor analyses demonstrated that the fit of the three-factor LCQ was acceptable at baseline (Supplementary Results, Supplementary Table S2).

### Consistency and stability of LCQ

Internal consistency (Cronbach’s α) demonstrated good to excellent reliability at baseline and at weeks 4 and 8 for the LCQ total (Cronbach’s α = 0.884–0.940), physical domain (Cronbach’s α = 0.703–0.770), psychological domain (Cronbach’s α = 0.814–0.914), and social domain (Cronbach’s α = 0.777–0.892) scores.

When using a cough severity VAS score change of ⩽10 mm from baseline to week 4 to define participants as *stable*, test–retest reliability of the LCQ scores was good (ICC of 0.75–0.80; Table 1). A similar pattern was found when using awake cough frequency (i.e., change of ⩽10% from baseline to week 4) and *no change* on the PGIC at week 4 to define *stable* disease (Supplementary Table S3).

**Table 1.** Test–retest reliability (reproducibility) of LCQ scores: participants reporting ⩽10-mm change on the cough severity VAS from baseline to week 4 (*n* = 61).

LCQ domain	Baseline, mean (SD)	Week 4, mean (SD)	Difference^a^	*p* value	Pearson’s *r*^b^	ICC
Total score	12.5 (3.41)	13.6 (3.79)	1.1	<0.0001	0.84	0.80
Physical	4.7 (1.02)	5.0 (1.03)	0.2	0.0069	0.77	0.75
Psychological	4.0 (1.38)	4.4 (1.47)	0.3	0.0026	0.82	0.80
Social	3.7 (1.34)	4.3 (1.61)	0.5	<0.0001	0.81	0.75

ICC, intraclass correlation coefficient; LCQ, Leicester Cough Questionnaire; SD, standard deviation; VAS, visual analogue scale.

a Difference = week 4 − baseline for daily score.

b Pearson’s product–moment correlation.

### Validity and responsiveness

Correlations between the LCQ total and domain scores *versus* related measures were low to moderate in magnitude (Table 2). Stronger associations were demonstrated at week 4, supporting convergent validity of the LCQ total and domain scores (Supplementary Table S4). At week 4, the LCQ total score change from baseline correlated moderately with percentage changes from baseline in awake and 24-h objective cough frequency (Supplementary Table S5).

**Table 2.** Pearson’s correlations between LCQ scores and conceptually related measures at baseline.

Parameter	LCQ score at baseline^a^			
	Total	Physical	Psychological	Social
CSD				
Total score	−0.64	−0.59	−0.48	−0.60
Frequency	−0.59	−0.52	−0.47	−0.55
Intensity	−0.59	−0.53	−0.44	−0.57
Disruption	−0.61	−0.61	−0.43	−0.58
Cough severity VAS	−0.41	−0.29	−0.38	−0.40

CSD, Cough Severity Diary; LCQ, Leicester Cough Questionnaire; VAS, visual analogue scale.

a Pearson’s correlation coefficients reported; all are *p* < 0.0001.

In support of known-groups validity, the LCQ total and domain scores were lowest (indicating worse cough-specific quality of life) in participants in the highest CSD score group (indicating worse cough severity) and increased with improving levels of CSD scores (*p* < 0.0001; Table 3). The same pattern was found when investigating known-groups validity across tertiles of CSD scores at week 4 and tertiles of awake cough frequency at baseline and week 4 (Supplementary Table S6).

**Table 3.** Known-groups validity: LCQ score at baseline by CSD total score groups at baseline.^a^

LCQ domain	CSD tertile Group 1		CSD tertile Group 2		CSD tertile Group 3		Overall *F* value	*p* value
	*N*	Mean (SE)	*N*	Mean (SE)	*N*	Mean (SE)		
Total score	76	13.5 (0.30)	85	11.7 (0.29)	78	9.9 (0.30)	35.68	<0.0001
Physical	76	5.0 (0.10)	85	4.6 (0.10)	78	3.7 (0.10)	40.20	<0.0001
Psychological	76	4.3 (0.13)	85	3.6 (0.12)	78	3.2 (0.13)	16.41	<0.0001
Social	76	4.3 (0.13)	85	3.5 (0.12)	78	3.0 (0.12)	29.82	<0.0001

CSD, Cough Severity Diary; LCQ, Leicester Cough Questionnaire; SE, standard error.

a Participants were stratified into tertiles using sample distribution according to CSD score at baseline. Group 1 represents participants with the lowest CSD scores (i.e., lowest cough severity), whereas group 3 represents those with the highest CSD scores (i.e., highest cough severity).

Responsiveness of LCQ total and domain scores was supported when using the PGIC at week 4 to define responders. Participants with a PGIC score of 1 or 2 (*very much improved*, *much improved*) had the greatest mean improvement on LCQ total score from baseline to week 4 and a large effect size (Table 4). Mean change in the LCQ total score was smaller with each subsequent PGIC category, with corresponding smaller effect sizes for each group. A similar pattern was found for each of the LCQ domains (Supplementary Table S7).

**Table 4.** Responsiveness of LCQ scores: LCQ total from baseline to week 4 by PGIC category and awake cough frequency.

Category	*N*	Baseline, mean (SD)	Week 4, mean (SD)	Mean score change^a^		Effect size^b^
				Difference	Range	
PGIC score						
1 or 2	87	12.0 (2.94)	17.5 (2.57)	5.6	−4.7 to 12.3	1.9
3	78	11.9 (2.99)	14.2 (2.65)	2.3	−2.2 to 6.7	0.8
4	61	11.4 (2.75)	11.9 (2.89)	0.5	−3.2 to 7.0	0.2
5	5	9.6 (3.91)	9.1 (3.22)	−0.6	−1.8 to 1.3	−0.2
6 or 7	4	12.7 (3.28)	10.2 (3.73)	−2.6	−4.0 to −1.9	−0.8
Awake cough frequency						
⩾30% reduction	126	11.9 (2.74)	16.0 (3.27)	4.1	−4.7 to 12.3	1.5
<30% reduction	102	11.5 (3.22)	12.9 (3.38)	1.3	−4.0 to 10.2	0.4
⩾50% reduction	79	11.7 (2.89)	16.9 (2.94)	5.2	−4.7 to 12.3	1.8
<50% reduction	149	11.8 (3.01)	13.4 (3.46)	1.6	−4.0 to 10.8	0.5
⩾70% reduction	52	11.7 (2.70)	17.5 (3.06)	5.8	−4.7 to 12.3	2.2
<70% reduction	176	11.8 (3.04)	13.8 (3.39)	2.0	−4.0 to 10.8	0.7
Reduction ⩾0.30 SD	118	11.5 (2.70)	15.6 (3.48)	4.2	−4.7 to 12.3	1.5
Reduction <0.30 SD	110	12.1 (3.21)	13.5 (3.57)	1.5	−4.0 to 10.2	0.5

LCQ, Leicester Cough Questionnaire; PGIC, patient global impression of change; SD, standard deviation.

a Calculated as week 4 − baseline.

b Calculated as score difference/SD of baseline score.

When using various thresholds of change in awake cough frequency to define responders (i.e., ⩾30%, ⩾50%, ⩾70%, and ⩾0.30 SD reductions), mean score changes and effect sizes for the LCQ total score were always larger for those considered responders *versus* nonresponders (Table 4). Similar results were observed for 24-h cough frequency (Supplementary Table S8).

### Clinically meaningful change threshold

Distribution-based estimates of one-half SD were 1.51 (total), 0.51 (physical), 0.61 (psychological), and 0.61 (social). The SEs of measurement estimates were 1.51 (total), 0.42 (physical), 0.60 (psychological), and 0.85 (social).

Using an anchor-based approach for estimating a clinically meaningful change threshold, the mean (SD) LCQ score change from baseline to week 4 for participants who reported themselves as *minimally improved* on the PGIC (score of 3) was 2.3 (1.9) for the total score and ranged from 0.5 to 0.9 (0.6–0.9) for domain scores (Table 5).

**Table 5.** Mean change in LCQ total and domain scores from baseline to week 4 by PGIC group at week 4.

PGIC score	Change in LCQ			
	Total score, mean (SD)	Physical score, mean (SD)	Psychological score, mean (SD)	Social score, mean (SD)
1 or 2	5.6 (3.5)	1.4 (1.1)	2.0 (1.4)	2.2 (1.4)
3	2.3 (1.9)	0.5 (0.6)	0.9 (0.9)	0.9 (0.8)
4	0.5 (1.8)	0.1 (0.6)	0.2 (0.9)	0.2 (1.0)
5	−0.6 (1.2)	−0.1 (0.5)	−0.1 (0.5)	−0.4 (0.5)
6 or 7	−2.6 (1.0)	−0.2 (0.7)	−0.8 (0.4)	−1.6 (0.4)

LCQ, Leicester Cough Questionnaire; PGIC, patient global impression of change; SD, standard deviation.

**Figure 1.**
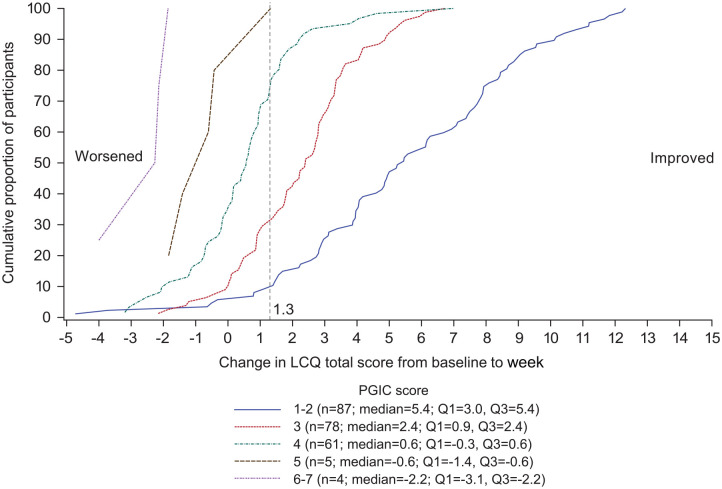
CDF curve: change in LCQ total score from baseline (day 0) to week 4 (day 28) by PGIC. CDF, cumulative distribution function; LCQ, Leicester Cough Questionnaire; PGIC, patient global impression of change.

**Figure 2.**
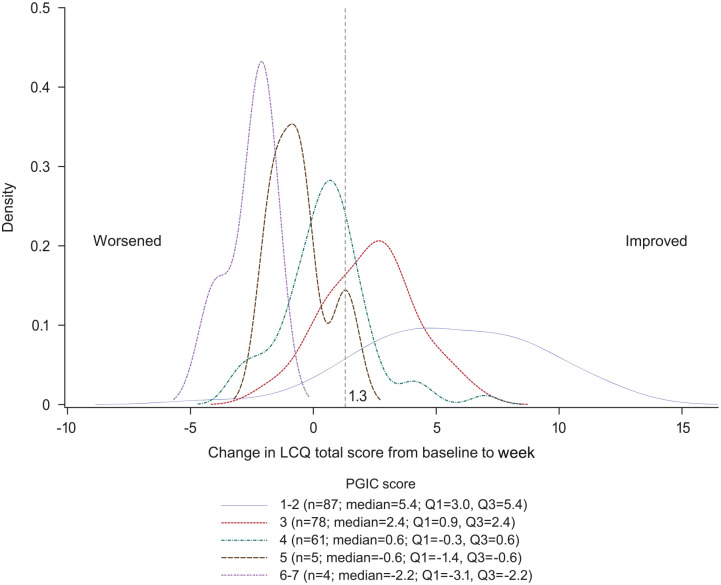
PDF curve: change in LCQ total score from baseline (day 0) to week 4 (day 28) by PGIC. LCQ, Leicester Cough Questionnaire; PDF, probability density function; PGIC, patient global impression of change.

The eCDF (Figure 1) and ePDF (Figure 2) curves show separation of both curves for each PGIC rating across a range of LCQ total score changes from baseline to week 4, including at the 1.3-point threshold previously published as a meaningful change.

To further assess the threshold values for change in LCQ total and domain scores, ROC curves were evaluated to identify change scores with the best sensitivity and specificity for predicting participants scoring a 1 to 3 *versus* 4 to 7 (Supplementary Figure S2A–D) or 1 to 2 *versus* 3 to 7 (Supplementary Figure S3) on the PGIC. Results demonstrated that no single LCQ threshold maximized sensitivity and specificity for a PGIC of 1 to 3 at week 4 (Table 6). For PGIC ratings of 1 to 3, sensitivity for the previously published threshold of a ⩾1.3-point change on the LCQ total score was among the highest, whereas specificity was maximized at a threshold of ⩾2.7. The Youden index ranged from 0.56 to 0.61 between these LCQ thresholds, representing differing trade-offs between maximizing sensitivity and specificity. The ROC curves using PGIC categories of 1 and 2 showed support for higher thresholds of change in LCQ, with the Youden index being highest at a ⩾2.9 change on the LCQ total score (Supplementary Table S9). For the LCQ domains, Youden index for PGIC of 1 to 3 at week 4 was maximized at thresholds between 0.8 and 1.0 (Table 6).

**Table 6.** ROC curve analysis for LCQ score thresholds predictive of PGIC of 1 to 3 at week 4.

LCQ score-change threshold	Sensitivity	Specificity	Positive predictive value	Negative predictive value	Youden index
Total score					
⩾1.0	0.83	0.70	0.87	0.64	0.53
⩾1.3	0.81	0.76	0.89	0.62	0.56
⩾1.5	0.78	0.81	0.91	0.61	0.60
⩾1.7	0.76	0.86	0.93	0.60	0.61
⩾2.0	0.72	0.89	0.94	0.57	0.61
⩾2.3	0.68	0.91	0.95	0.55	0.60
⩾2.7	0.64	0.94	0.96	0.53	0.59
⩾3.0	0.56	0.94	0.96	0.47	0.50
Physical					
⩾0.8	0.49	0.94	0.95	0.44	0.43
⩾0.9	0.44	0.94	0.95	0.42	0.39
Psychological					
⩾0.9	0.62	0.89	0.93	0.50	0.51
⩾1.0	0.62	0.89	0.93	0.50	0.51
Social					
⩾0.8	0.65	0.86	0.92	0.51	0.51
⩾0.9	0.65	0.86	0.92	0.51	0.51
⩾1.0	0.65	0.86	0.92	0.51	0.51

LCQ, Leicester Cough Questionnaire; PGIC, patient global impression of change; ROC, receiver operating characteristic.

Because an LCQ total score change of ⩾1.7 was the lowest score-change threshold that maximized the Youden index (Table 6), this threshold was used to define an LCQ response to evaluate the relationship between PGIC scores and LCQ responder status. Of those who had a total PGIC score of 1 or 2 (*very much improved, much improved*), 85% were considered LCQ responders (Table 7).

**Table 7.** Proportion of responders based on LCQ total score by PGIC group at week 4.

PGIC score, *n* (%)	Responder defined by LCQ total score change of ⩾1.7 (baseline to week 4)	
	Responder	Nonresponder
1 or 2	74 (85.1)	13 (14.9)
3	51 (65.4)	27 (34.6)
4	10 (16.4)	51 (83.6)
5	0	5 (100.0)
6 or 7	0	4 (100.0)

LCQ, Leicester Cough Questionnaire; PGIC, patient global impression of change.

## Discussion

This analysis using data from a phase IIb clinical trial that included more than 220 participants confirms the psychometric properties of the LCQ in a population of participants with RCC or UCC. These results support the use of the LCQ in this population as a valid, reliable, and responsive measure to assess the impact of CC on HRQOL. These results also support the previously established threshold for defining a minimum clinically meaningful within-patient change of ⩾1.3 points on the LCQ total score and explore the potential for higher thresholds to identify participants with greater improvements in HRQOL.

One important attribute of any PRO is reliability, or the extent to which an instrument yields the same score each time it is administered when the underlying construct measured has not changed. Internal consistency of the LCQ total score was good to excellent (Cronbach’s α between 0.884 and 0.940).^29^ When score changes were calculated between the initial LCQ scores (baseline) and retest LCQ scores (week 4) among a subset of participants categorized as *stable* during that time frame based on changes in awake cough frequency, cough severity VAS, and PGIC, test–retest reliability of the LCQ total and domain scores indicated moderate to good reliability.

A second important attribute is validity, or the extent to which the LCQ measures the impact of cough. Validity was evaluated through LCQ correlation with instruments measuring similar or related constructs. The LCQ total and domain scores demonstrated convergent validity with moderate to high correlations (at week 4) with other similar cough constructs including the CSD and cough severity VAS (*p* < 0.0001). The LCQ also distinguished between groups of participants with different disease severity, as determined by CSD scores and awake/24-h cough frequency.

A third important attribute of PROs, responsiveness, was also assessed and confirmed for the LCQ. Participants who self-reported as being *very much improved* or *much improved* (PGIC of 1 or 2) had the greatest improvements in mean LCQ scores. Mean LCQ improvements were also greater in participants who had the highest reductions in awake cough frequency. Taken together, these findings provide strong support for the responsiveness of the LCQ in individuals with RCC or UCC.

These results are generally consistent with the previously established threshold for defining a minimum clinically meaningful within-patient change of ⩾1.3 points on the LCQ total score in patients with CC.^21^ We considered findings from multiple methods of triangulating the meaningful change threshold. The mean LCQ total score changes for participants reporting *no change* (0.5-point increase) and *minimally improved* (2.3-point increase) on the PGIC would suggest that a responder threshold would be between these two values. Distribution-based estimates (one-half of the SD and SE of measurement) both pointed to values of 1.51 for a potential LCQ responder threshold. Finally, ROC curve analyses supported multiple potential responder definitions, with competing changes in sensitivity and specificity resulting in little difference in Youden index for thresholds between 1.3- and 2.3-point changes in the LCQ total score. Further research in a broader patient population may be needed to refine the threshold for defining responders based on the LCQ total score, dependent on whether sensitivity, specificity, or both are of greatest importance for the context of use. For example, a higher threshold of a 2.3-point change in the LCQ total score may be useful to identify patients experiencing a large degree of overall improvement (i.e., PGIC categories of *much improved* or *very much improved*). Conversely, in the context of a randomized controlled trial, where the goal is to identify the smallest degree of change perceived as an improvement by patients, lower thresholds corresponding to *minimally improved* PGIC ratings or where sensitivity and specificity are maximized may be more appropriate for defining responders to treatment. Although the idea of reporting a single threshold to define meaningful within-patient change is tempting, the eCDF and ePDF curves support the fact that there are a range of thresholds that could be considered meaningful for any given patient. Overall, these data support a minimum meaningful change threshold of 1.3 points for the LCQ total score; however, a range of thresholds from 1.3 to 2.3 points could also be considered depending on the context of use. Although a 1.3- to 2.3-point change could be considered small relative to the LCQ total score range, a small degree of meaningful change is not inconsistent with other respiratory scales (e.g., a 4-point change on the 100-point St George’s Respiratory Questionnaire is considered the minimum clinically important difference).^30^

From a patient or clinician perspective, the ultimate goal of therapy is to reduce the impact of cough and improve quality of life, which supports the LCQ as an essential endpoint in clinical trials. Regulators often prefer the use of objective primary endpoints in clinical trials to ensure that the efficacy of therapy is indeed due to a reduction in cough and not other mechanisms (e.g., altered perception). The concept that subjective assessments and objectively measured cough counting reveal different facets of the clinical cough is important, and a combination of these tools is critical for understanding the efficacy of a CC therapy. Further work is necessary to better understand the relative importance of cough frequency and PROs from a patient perspective.

There were limitations to this analysis. First, participants were enrolled in a clinical trial and pooled regardless of the treatment assignment. Although this has several advantages for evaluating psychometric properties of the LCQ in participants whose cough is expected to improve, it may also diverge from previous validation efforts in patients whose cough follows a more stable or natural progression. Second, the reliability of an instrument is best assessed when a gold standard for measuring change in a construct is available. In the context of CC, no such gold standard exists; therefore, although reliability may have been somewhat lower than expected, this could be due to the lack of an appropriate metric for defining a *stable* population. Third, this trial only enrolled participants in the United States and the United Kingdom, potentially limiting the global generalizability of the results. Fourth, the measures captured in the phase IIb study did not directly capture meaningfulness of changes according to study participants; thus, the threshold was inferred on the basis of PGIC scores, which assessed improvement rather than meaningfulness. Finally, the RCC and UCC populations may differ from general CC populations previously studied and, considering trial eligibility criteria, it is unclear how the minimum clinically important change obtained in this study may apply to a more general population of individuals who have RCC or UCC related to other conditions (e.g., chronic obstructive pulmonary disease, cystic fibrosis).^8,21^ Regardless, participants in this trial underwent a guidelines-based approach to diagnosis and treatment of any potential comorbid conditions contributing to CC, which is expected to be consistent with the diagnosis of RCC or UCC in clinical practice. They were also required to have a cough severity VAS ⩾40 mm and a cough lasting for ⩾1 year at screening for inclusion.^22^ Thus, although this population may have had a more severe, long-lasting cough than those in a real-world setting, the participants enrolled in this trial may be similar to patients likely to seek treatment.

These limitations should be considered within the context of the strengths of this study. There are numerous advantages of performing these analyses in a clinical trial setting, including the large sample size, variety of outcomes collected, and relatively small amounts of missing data. This also allowed for the evaluation of LCQ psychometric properties using objectively measured cough frequency, which is not commonly available outside of clinical trial settings. Finally, the protocol-based approach of enrolling participants and verifying eligibility provides consistency and transparency regarding the definition of this population.

## Conclusion

This analysis supports the reliability and validity of the LCQ. The LCQ demonstrates good psychometric properties and has been found to be highly responsive in participants with RCC or UCC within a clinical trial setting. The proposed meaningful change threshold of the LCQ total score can be used to better understand and interpret clinical trial results and may help identify responders and nonresponders to treatment. Clinicians may consider a 1.3-point increase as a minimum meaningful change for patients in the LCQ total score, although a broader range of thresholds from 1.3- to 2.3-point increases could be considered depending on the context of use.

## Supplemental Material

sj-pdf-1-tar-10.1177_17534666221099737 – Supplemental material for Leicester Cough Questionnaire validation and clinically important thresholds for change in refractory or unexplained chronic cough Click here for additional data file. Supplemental material, sj-pdf-1-tar-10.1177_17534666221099737 for Leicester Cough Questionnaire validation and clinically important thresholds for change in refractory or unexplained chronic cough by Allison Martin Nguyen, Jonathan Schelfhout, David Muccino, Elizabeth D. Bacci, Carmen La Rosa, Margaret Vernon and Surinder S. Birring in Therapeutic Advances in Respiratory Disease

sj-pdf-2-tar-10.1177_17534666221099737 – Supplemental material for Leicester Cough Questionnaire validation and clinically important thresholds for change in refractory or unexplained chronic cough Click here for additional data file. Supplemental material, sj-pdf-2-tar-10.1177_17534666221099737 for Leicester Cough Questionnaire validation and clinically important thresholds for change in refractory or unexplained chronic cough by Allison Martin Nguyen, Jonathan Schelfhout, David Muccino, Elizabeth D. Bacci, Carmen La Rosa, Margaret Vernon and Surinder S. Birring in Therapeutic Advances in Respiratory Disease

sj-pdf-3-tar-10.1177_17534666221099737 – Supplemental material for Leicester Cough Questionnaire validation and clinically important thresholds for change in refractory or unexplained chronic cough Click here for additional data file. Supplemental material, sj-pdf-3-tar-10.1177_17534666221099737 for Leicester Cough Questionnaire validation and clinically important thresholds for change in refractory or unexplained chronic cough by Allison Martin Nguyen, Jonathan Schelfhout, David Muccino, Elizabeth D. Bacci, Carmen La Rosa, Margaret Vernon and Surinder S. Birring in Therapeutic Advances in Respiratory Disease
