# Supplementary material for: Leicester Cough Questionnaire validation and clinically important thresholds for change in refractory or unexplained chronic cough
Source: Ther Adv Respir Dis. 2022 May 25;16:17534666221099737. doi: 10.1177/17534666221099737 (PMC9149626; doi:10.1177/17534666221099737)

SUPPLEMENTARY FIGURES

Supplementary Figure S1. Leicester Cough Questionnaire items by domain. Numbers represent corresponding question numbers (or item numbers) in questionnaire.

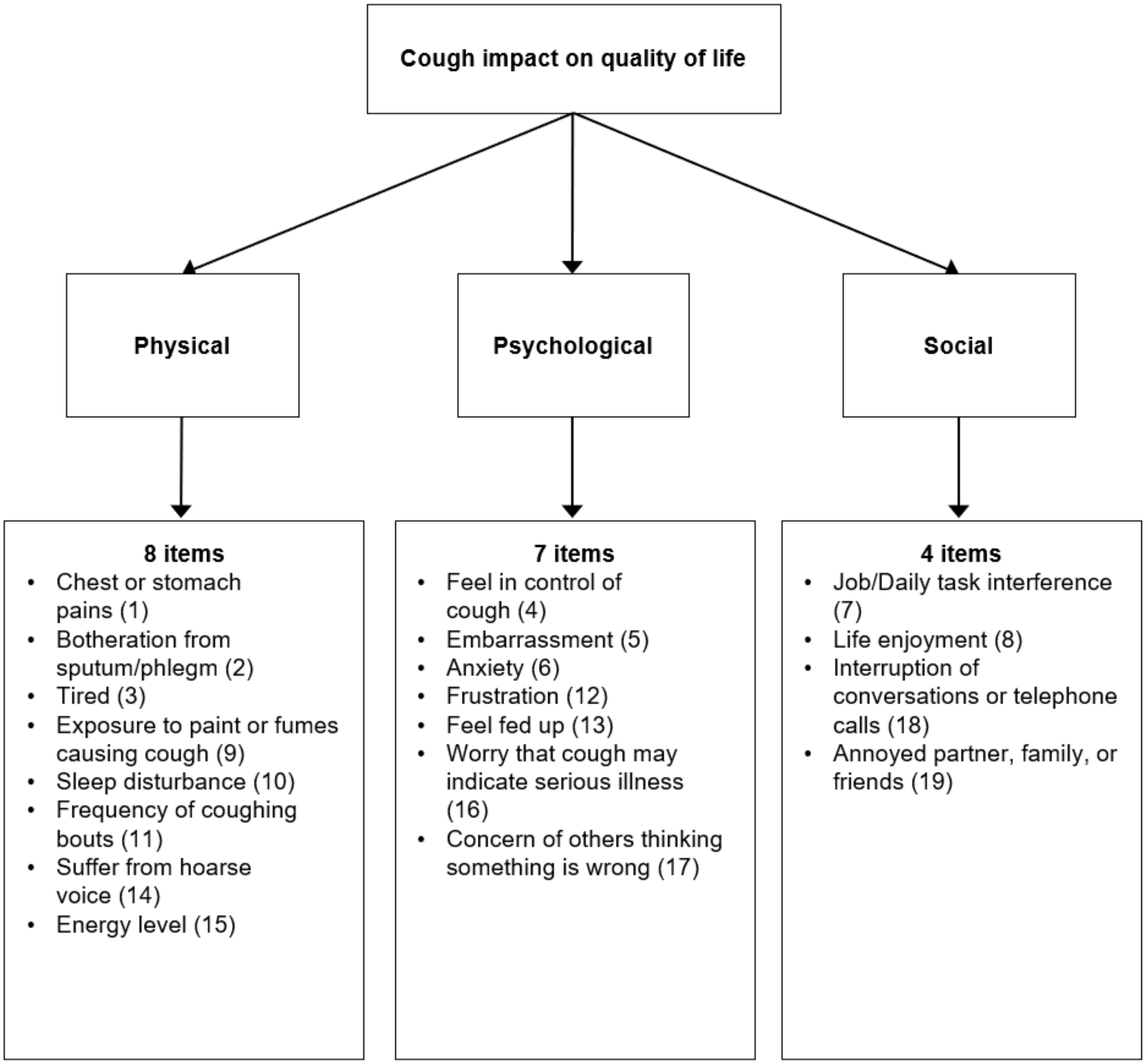

Supplementary Figure S2. ROC curves for the LCQ **(A)** total, **(B)** physical, **(C)** psychological, and **(D)** social score changes for predicting global improvements based on the PGIC (scores of 1 to 3) at Week 4. LCQ, Leicester Cough Questionnaire; PGIC, patient global impression of change; ROC, receiver operating characteristic.

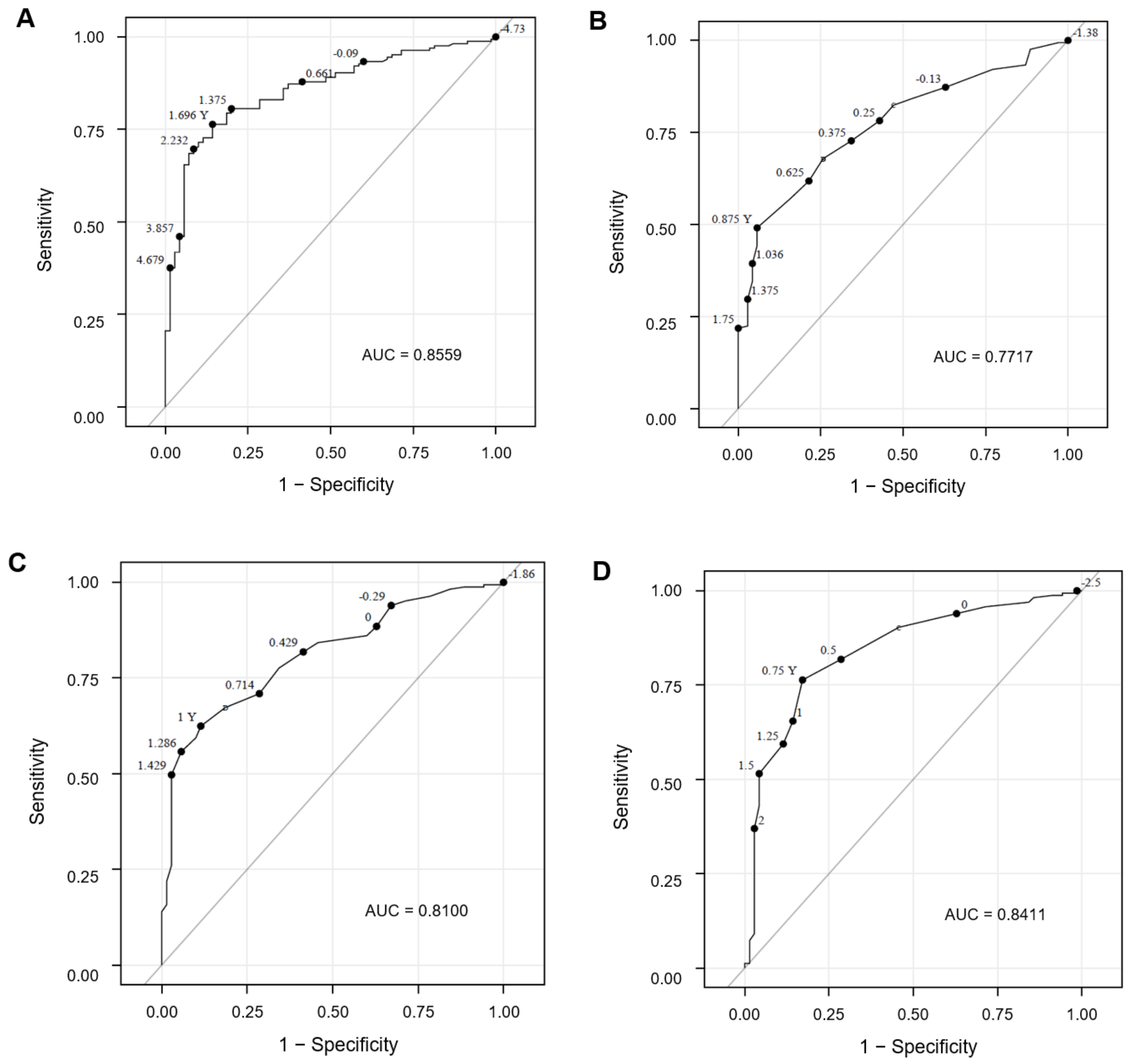

Supplementary Figure S3. ROC curve for the LCQ total score change for predicting global improvements based on the PGIC (scores of 1 to 2) at Week 4. Leicester Cough Questionnaire; PGIC, patient global impression of change; ROC, receiver operating characteristic.

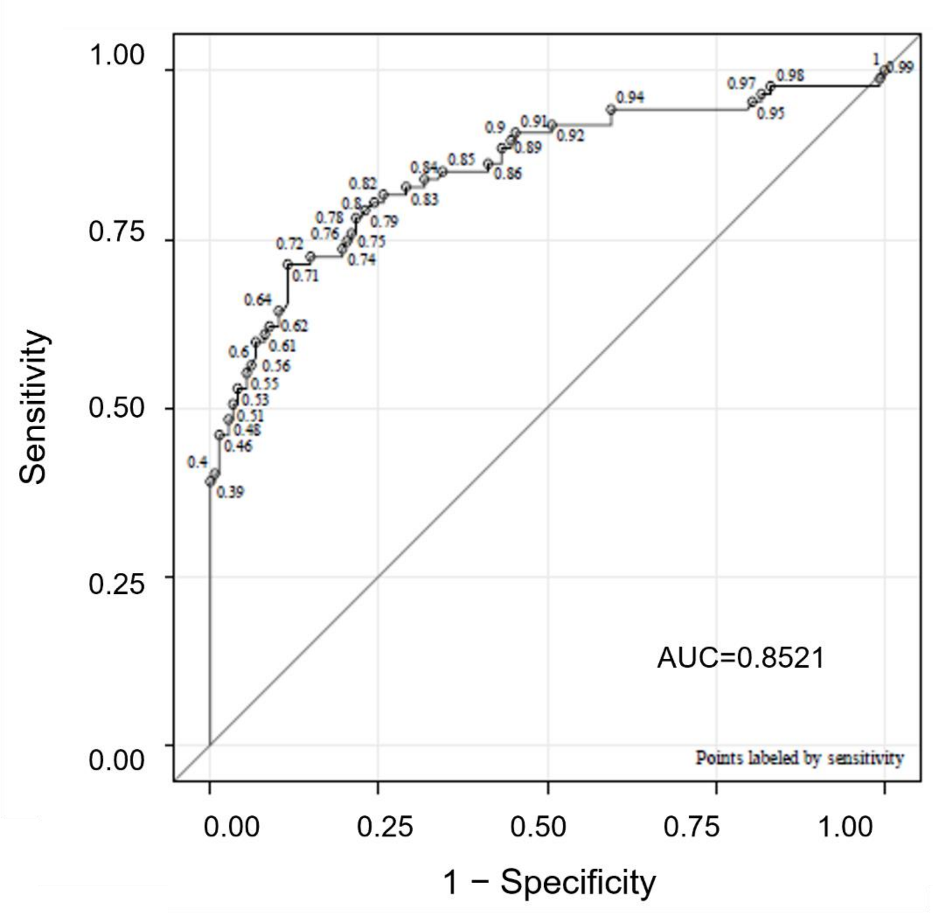

Supplement: sj-pdf-1-tar-10.1177_17534666221099737 – Supplemental material for Leicester Cough Questionnaire validation and clinically important thresholds for change in refractory or unexplained chronic cough [file sj-pdf-1-tar-10.1177_17534666221099737.pdf]
