# Supplementary material for: Leicester Cough Questionnaire validation and clinically important thresholds for change in refractory or unexplained chronic cough
Source: Ther Adv Respir Dis. 2022 May 25;16:17534666221099737. doi: 10.1177/17534666221099737 (PMC9149626; doi:10.1177/17534666221099737)
Supplement: sj-pdf-2-tar-10.1177_17534666221099737 – Supplemental material for Leicester Cough Questionnaire validation and clinically important thresholds for change in refractory or unexplained chronic cough [file sj-pdf-2-tar-10.1177_17534666221099737.pdf]

SUPPLEMENTARY METHODS

Patient-reported outcomes for Leicester Cough Questionnaire validation

Several patient-reported outcome measures were used for Leicester Cough Questionnaire (LCQ) validation. The 7-item Cough Severity Diary (CSD) was designed to capture the patient's assessment of cough severity across 3 domains of frequency, intensity, and disruption on an 11-point scale ranging from 0 to 10, with a recall period of today. The CSD is scored by summing daily scores across 7 days to calculate mean weekly total and domain scores, with higher scores reflecting greater severity. The CSD was completed daily throughout the study. The single-item cough severity visual analog scale (VAS) rates the patient's perception of cough severity over the past 24 hours using a 100-mm scale ranging from 0 (*no cough*) to 100 (*worst cough*) and was administered at baseline and Weeks 4, 8, and 12. The single-item patient global impression of change (PGIC), a 7-point scale depicting a patient's rating of overall improvement ranging from 1 (*very much improved*) to 7 (*very much worse*), was included at Weeks 4, 8, and 12 to capture the patient's perception of overall change in cough since baseline. Finally, cough frequency (number of coughs per hour) was captured at baseline and Weeks 4, 8, and 12 using the VitaloJAK® (Vitalograph, Buckingham, UK). The VitaloJAK is a wearable, 24-hour cough-recording device, for which validation has been previously published (McGuinness et al. *Thorax.* 2012;67[suppl 2]:A131. Abstract P159). The VitaloJAK was used to obtain measures of awake and 24-hour cough frequency.

Domains and confirmatory factor analyses

Several fit statistics were used to assess the adequacy of the structural-equation-modeling approach, including a comparative-fit index (CFI) ≥0.9, a standardized root mean square residual (SRMR) <0.1 (Kline. *Educ Res Eval.* 2013;19:204-222), and a root mean square error of approximation (RMSEA) <0.08 (Breivik and Olsson. In: Cudeck et al, eds. *Structural Equation Modeling: Present and Future.* 2001:169-194). Adequacy of fit was also

assessed by examining modification indices. Factor loadings of 0.40 were considered acceptable.

SUPPLEMENTARY RESULTS

Domains and confirmatory factor analyses

Individual-item analyses of the LCQ were performed at baseline and Week 4. A nonhierarchical 3-factor structure for the LCQ converged successfully at baseline. Confirmatory factor analyses were performed at baseline and Week 4 using a structural-equation-modeling approach to confirm scaling and fit of proposed domains. The overall fit of the 3-factor LCQ (including physical, psychological, and social factors) was found to be acceptable at baseline according to the CFI and SRMR values (0.929 and 0.053, respectively; $\chi^2(144) = 268.05$, $P<0.0001$). Root mean square error of approximation (0.0058) was also acceptable. Similar results were found at Week 4. For the models that converged successfully, item factor loadings were acceptable (>0.40), except for 2 items that queried physical symptoms (botheration from sputum/phlegm [item 2; baseline and Week 4], hoarse voice [item 14; baseline only]) and 2 items that queried social symptoms (interruption of conversations/phone calls [item 18; Week 4 only], annoyed partner/family/friends [item 19; baseline only]).

A second series of hierarchical confirmatory factor analyses was also conducted, including all 3 LCQ domain factors and the LCQ total score. At baseline, the model failed to converge, rendering interpretation of these values difficult. However, at Week 4, the model converged successfully, and the fit of the model was acceptable (CFI = 0.925; SRMR = 0.047; RMSEA = 0.078). All factor loadings except for items 1 and 2 in the physical domain (chest or stomach pains [item 1], botheration from sputum/phlegm [item 2]) were also acceptable.

The overall fit of the nonhierarchical 3-factor LCQ (including physical, psychological, and social factors) was found to be acceptable at baseline and Week 4, with most item factor loadings also acceptable at baseline and Week 4; however, low factor loadings were found for a few items. Of note, in the series of hierarchical confirmatory factor analyses, the model

successfully converged at Week 4, but not baseline. A suspected reason for nonconvergence is the presence of very strong item loadings that load across all factors (ie, cross-loading items).
