# Supplementary material for: Leicester Cough Questionnaire validation and clinically important thresholds for change in refractory or unexplained chronic cough
Source: Ther Adv Respir Dis. 2022 May 25;16:17534666221099737. doi: 10.1177/17534666221099737 (PMC9149626; doi:10.1177/17534666221099737)
Supplement: sj-pdf-3-tar-10.1177_17534666221099737 – Supplemental material for Leicester Cough Questionnaire validation and clinically important thresholds for change in refractory or unexplained chronic cough [file sj-pdf-3-tar-10.1177_17534666221099737.pdf]

SUPPLEMENTARY TABLES

Supplementary Table S1. Leicester Cough Questionnaire Item-Level Descriptive Statistics at Baseline and Week 4[a]

Measures	Baseline (N=253)	Week 4 (N=236)
**Item 1**		
Mean (SD)	5.7 (1.6)	6.4 (1.2)
Range	1.0, 7.0	1.0, 7.0
Floor, n (%)	8 (3.2)	3 (1.3)
Ceiling, n (%)	118 (46.6)	161 (68.2)
**Item 2**		
Mean (SD)	4.3 (1.8)	4.8 (1.8)
Range	1.0, 7.0	1.0, 7.0
Floor, n (%)	18 (7.1)	10 (4.2)
Ceiling, n (%)	32 (12.6)	53 (22.5)
**Item 3**		
Mean (SD)	4.3 (1.8)	5.3 (1.8)
Range	1.0, 7.0	1.0, 7.0
Floor, n (%)	23 (9.1)	8 (3.4)
Ceiling, n (%)	34 (13.4)	80 (33.9)
**Item 4**		
Mean (SD)	3.2 (1.7)	4.5 (1.8)
Range	1.0, 7.0	1.0, 7.0
Floor, n (%)	44 (17.4)	20 (8.5)
Ceiling, n (%)	11 (4.3)	38 (16.1)
**Item 5**		
Mean (SD)	4.7 (1.8)	5.6 (1.6)
Range	1.0, 7.0	1.0, 7.0
Floor, n (%)	14 (5.5)	5 (2.1)
Ceiling, n (%)	50 (19.8)	94 (39.8)
**Item 6**		
Mean (SD)	4.4 (1.6)	5.3 (1.6)
Range	1.0, 7.0	1.0, 7.0
Floor, n (%)	16 (6.3)	5 (2.1)
Ceiling, n (%)	25 (9.9)	75 (31.8)
**Item 7**		
Mean (SD)	3.6 (1.5)	4.9 (1.6)
Range	1.0, 7.0	1.0, 7.0
Floor, n (%)	21 (8.3)	10 (4.2)
Ceiling, n (%)	10 (4.0)	45 (19.1)
**Item 8**		
Mean (SD)	4.7 (2.2)	5.4 (2.0)
Range	1.0, 7.0	1.0, 7.0
Floor, n (%)	29 (11.5)	14 (5.9)
Ceiling, n (%)	90 (35.6)	115 (48.7)
**Item 9**		
Mean (SD)	4.4 (1.9)	5.4 (1.5)
Range	1.0, 7.0	1.0, 7.0
Floor, n (%)	25 (9.9)	6 (2.5)
Ceiling, n (%)	45 (17.8)	70 (29.7)

**Item 10**		
Mean (SD)	3.5 (1.3)	4.3 (1.5)
Range	1.0, 7.0	1.0, 7.0
Floor, n (%)	17 (6.7)	12 (5.1)
Ceiling, n (%)	5 (2.0)	15 (6.4)
**Item 11**		
Mean (SD)	3.2 (1.6)	4.5 (1.9)
Range	1.0, 7.0	1.0, 7.0
Floor, n (%)	45 (17.8)	22 (9.3)
Ceiling, n (%)	8 (3.2)	39 (16.5)
**Item 12**		
Mean (SD)	3.3 (1.8)	4.7 (2.0)
Range	1.0, 7.0	1.0, 7.0
Floor, n (%)	52 (20.6)	19 (8.1)
Ceiling, n (%)	16 (6.3)	64 (27.1)
**Item 13**		
Mean (SD)	4.9 (1.8)	5.5 (1.7)
Range	1.0, 7.0	1.0, 7.0
Floor, n (%)	11 (4.3)	6 (2.5)
Ceiling, n (%)	69 (27.3)	102 (43.2)
**Item 14**		
Mean (SD)	5.5 (1.9)	6.1 (1.4)
Range	1.0, 7.0	1.0, 7.0
Floor, n (%)	13 (5.1)	3 (1.3)
Ceiling, n (%)	115 (45.5)	140 (59.3)
**Item 15**		
Mean (SD)	3.3 (1.8)	4.4 (2.0)
Range	1.0, 7.0	1.0, 7.0
Floor, n (%)	54 (21.3)	29 (12.3)
Ceiling, n (%)	16 (6.3)	41 (17.4)
**Item 16**		
Mean (SD)	3.1 (1.4)	4.2 (1.7)
Range	1.0, 7.0	1.0, 7.0
Floor, n (%)	35 (13.8)	12 (5.1)
Ceiling, n (%)	1 (0.4)	27 (11.4)
**Item 17**		
Mean (SD)	3.1 (1.7)	4.4 (1.9)
Range	1.0, 7.0	1.0, 7.0
Floor, n (%)	52 (20.6)	21 (8.9)
Ceiling, n (%)	13 (5.1)	45 (19.1)
**Item 18**		
Mean (SD)	2.7 (1.8)	3.9 (2.0)
Range	1.0, 7.0	1.0, 7.0
Floor, n (%)	79 (31.2)	35 (14.8)
Ceiling, n (%)	17 (6.7)	21 (8.9)
**Item 19**		
Mean (SD)	3.8 (1.6)	4.1 (1.7)
Range	1.0, 7.0	1.0, 7.0
Floor, n (%)	21 (8.3)	16 (6.8)
Ceiling, n (%)	10 (4.0)	12 (5.1)

Floor = minimum response >25%; ceiling = maximum response >25%. [a]All items range from 1 to 7.

Supplementary Table S2. Confirmatory Factor Loadings Using a 3-Factor Nonhierarchical Model of the LCQ at Baseline (N=253)

**LCQ item**	**Factor loadings**
Physical domain	
Item 1: Chest or stomach pains due to cough	0.489
Item 2: Bothered by sputum production with cough	0.188
Item 3: Tired because of cough	0.684
Item 9: Exposure to paint or fumes made me cough	0.618
Item 10: Cough disturbed sleep	0.628
Item 11: Coughing bouts per day	0.718
Item 14: Hoarse voice because of cough	0.339
Item 15: Had a lot of energy	0.757
Psychological domain	
Item 4: Felt in control of cough	0.766
Item 5: Felt embarrassed by cough	0.648
Item 6: Felt anxious by cough	0.585
Item 12: Felt frustrated by cough	0.755
Item 13: Felt fed up with cough	0.462
Item 16: Worried cough indicates serious illness	0.701
Item 17: Concerned other people think something is wrong because of cough	0.684
Social domain	
Item 7: Cough interfered with job or other daily tasks	0.717
Item 8: Cough interfered with overall enjoyment of life	0.421
Item 18: Cough interrupted conversation or telephone calls	0.337
Item 19: Cough annoyed partner, family, or friends	0.333

$\chi^2(144) = 268.05$ ($P<0.0001$); CFI = 0.929; RMSEA = 0.058 (90% CI: 0.047, 0.069); SRMR = 0.053.
CFI, comparative-fit index; LCQ, Leicester Cough Questionnaire; RMSEA, root mean square error of approximation; SRMR, standardized root mean square residual.

Supplementary Table S3. Test-Retest Reliability (Reproducibility) of LCQ Scores From Baseline to Week 4

LCQ domains	Baseline, mean (SD)	Week 4, mean (SD)	Difference[a]	*P* value	Pearson *r*[b]	ICC
≤10% change in awake cough frequency (n=32)						
Total score	12.1 (3.33)	12.7 (3.21)	0.7	0.1105	0.76	0.75
Physical	4.6 (1.04)	4.7 (0.93)	0.1	0.2045	0.84	0.83
Psychological	3.8 (1.28)	4.0 (1.26)	0.2	0.1188	0.77	0.76
Social	3.7 (1.38)	4.0 (1.32)	0.3	0.2391	0.52	0.51
No change on PGIC (n=61)						
Total score	11.4 (2.75)	11.9 (2.89)	0.5	0.0273	0.80	0.79
Physical	4.5 (0.90)	4.6 (0.87)	0.1	0.1391	0.79	0.78
Psychological	3.5 (1.12)	3.7 (1.14)	0.2	0.0917	0.70	0.69
Social	3.3 (1.22)	3.6 (1.28)	0.2	0.1095	0.65	0.64

ICC, intraclass correlation coefficient; LCQ, Leicester Cough Questionnaire; PGIC, patient global impression of change.
[a]Difference = Week 4 − baseline for daily score. [b]Pearson's product-moment correlation.

Supplementary Table S4. Pearson Correlations Between LCQ Scores and Conceptually Related Measures

	**Total**	**Physical**	**Psychological**	**Social**
**LCQ score at Week 4[a]**				
CSD				
Total score	-0.76	-0.69	-0.69	-0.73
Frequency	-0.75	-0.65	-0.71	-0.73
Intensity	-0.74	-0.69	-0.68	-0.71
Disruption	-0.65	-0.64	-0.57	-0.62
Cough severity VAS	-0.70	-0.60	-0.65	-0.69

CSD, Cough Severity Diary; LCQ, Leicester Cough Questionnaire; VAS, visual analog scale. [a]Pearson correlation coefficients reported; all are *P*<0.0001.

Supplementary Table S5. Pearson Correlations Between Change From Baseline in LCQ Total Score and Percentage Changes From Baseline in Awake and 24-Hour Objective Cough Frequency at Week 4 (n=228)

	**Percentage change in awake cough frequency[a]**	**Percentage change in 24-hour cough frequency[a]**
Change in LCQ total score from baseline to Week 4	-0.53	-0.53

LCQ, Leicester Cough Questionnaire. [a]Pearson correlation coefficients reported; both are *P*<0.0001.

Supplementary Table S6. Known-Groups Validity for LCQ Score at Week 4 by CSD Total Score at Week 4 and Awake Cough Frequency at Baseline and Week 4

	Tertile group 1		Tertile group 2		Tertile group 3			
**LCQ domain**	**N**	**Mean (SE)**	**N**	**Mean (SE)**	**N**	**Mean (SE)**	**Overall F value**	***P* value**
**CSD (Week 4)**								
Total score	76	17.4 (0.32)	75	14.9 (0.32)	68	11.7 (0.34)	74.68	<0.0001
Physical	76	5.8 (0.09)	75	5.2 (0.10)	68	4.4 (0.10)	50.43	<0.0001
Psychological	76	5.8 (0.13)	75	4.9 (0.13)	68	3.7 (0.14)	58.57	<0.0001
Social	76	5.8 (0.13)	75	4.7 (0.13)	68	3.6 (0.14)	67.44	<0.0001
**Awake cough frequency (baseline)**								
Total score	83	13.1 (0.31)	84	11.2 (0.31)	84	10.7 (0.31)	16.12	<0.0001
Physical	83	4.9 (0.11)	84	4.4 (0.10)	84	4.0 (0.10)	16.35	<0.0001
Psychological	83	4.2 (0.13)	84	3.5 (0.13)	84	3.4 (0.13)	10.73	<0.0001
Social	83	4.0 (0.13)	84	3.3 (0.13)	84	3.3 (0.13)	11.20	<0.0001
**Awake cough frequency (Week 4)**								
Total score	76	16.8 (0.36)	77	15.0 (0.36)	76	12.1 (0.36)	42.03	<0.0001
Physical	76	5.7 (0.10)	77	5.2 (0.10)	76	4.5 (0.10)	34.05	<0.0001
Psychological	76	5.6 (0.14)	77	4.9 (0.14)	76	3.9 (0.14)	34.94	<0.0001
Social	76	5.5 (0.15)	77	4.9 (0.15)	76	3.7 (0.15)	35.84	<0.0001

CSD, Cough Severity Diary; LCQ, Leicester Cough Questionnaire.

Supplementary Table S7. Responsiveness of LCQ Scores: LCQ Domains From Baseline to Week 4 by PGIC Category

PGIC score	N	Baseline, mean (SD)	Week 4, mean (SD)	Mean score change[a]		Effect size[b]
				Difference	Range	
LCQ physical						
1 or 2	87	4.4 (1.04)	5.8 (0.82)	1.4	-1.4, 4.0	1.3
3	78	4.5 (0.97)	5.0 (0.89)	0.5	-1.0, 2.4	0.5
4	61	4.5 (0.90)	4.6 (0.87)	0.1	-1.1, 1.6	0.1
5	5	3.4 (0.68)	3.3 (0.42)	-0.1	-0.6, 0.8	-0.1
6 or 7	4	4.8 (0.97)	4.7 (1.49)	-0.2	-1.0, 0.8	-0.2
LCQ psychological						
1 or 2	87	3.9 (1.19)	5.9 (0.91)	2.0	-1.9, 4.7	1.7
3	78	3.8 (1.26)	4.6 (1.13)	0.9	-1.3, 3.6	0.7
4	61	3.5 (1.12)	3.7 (1.14)	0.2	-1.6, 2.9	0.2
5	5	3.2 (1.60)	3.0 (1.54)	-0.1	-0.7, 0.6	-0.1
6 or 7	4	3.8 (1.38)	3.0 (1.25)	-0.8	-1.1, -0.1	-0.6
LCQ social						
1 or 2	87	3.7 (1.14)	5.8 (1.03)	2.2	-2.5, 5.3	1.9
3	78	3.7 (1.18)	4.6 (1.03)	0.9	-1.0, 3.0	0.8
4	61	3.3 (1.22)	3.6 (1.28)	0.2	-3.0, 4.8	0.2
5	5	3.1 (1.68)	2.8 (1.39)	-0.4	-1.0, 0.3	-0.2
6 or 7	4	4.1 (1.30)	2.5 (1.32)	-1.6	-2.0, -1.0	-1.3

LCQ, Leicester Cough Questionnaire; PGIC, patient global impression of change. [a]Calculated as Week 4 – baseline. [b]Calculated as score difference/SD of baseline score.

Supplementary Table S8. Responsiveness of LCQ Total Score: ANCOVA With LCQ Total Score Change From Baseline to Week 4 by 24-Hour Objective Cough Frequency Change at Week 4

		LCQ total score change	
**24-hour objective cough frequency change**	**N**	**Least-squares mean (SE)**	**Overall F value**
≥30% reduction			55.08*
Responder	126	4.3 (0.25)	—
Nonresponder	102	1.1 (0.28)	—
≥50% reduction			63.50*
Responder	79	5.2 (0.31)	—
Nonresponder	149	1.6 (0.22)	—
≥70% reduction			56.17*
Responder	50	5.9 (0.39)	—
Nonresponder	178	2.0 (0.21)	—
≥0.30 SD reduction			33.45*
Responder	123	3.9 (0.27)	—
Nonresponder	105	1.6 (0.29)	—

ANCOVA, analysis of covariance; LCQ, Leicester Cough Questionnaire. *P<0.0001.

Supplementary Table S9. ROC Curve Analysis for LCQ Total Score Thresholds Predictive of PGIC of 1 to 2 at Week 4

LCQ score-change threshold	Sensitivity	Specificity	Positive predictive value	Negative predictive value	Youden index
Total score					
≥1.0	0.92	0.47	0.51	0.91	0.39
≥1.3	0.91	0.52	0.53	0.91	0.43
≥1.5	0.89	0.56	0.54	0.89	0.45
≥1.7	0.85	0.59	0.55	0.87	0.44
≥2.0	0.85	0.64	0.58	0.88	0.49
≥2.1	0.85	0.65	0.59	0.88	0.50
≥2.2	0.84	0.66	0.59	0.88	0.50
≥2.3	0.83	0.68	0.61	0.87	0.51
≥2.4	0.83	0.70	0.62	0.87	0.53
≥2.5	0.83	0.71	0.63	0.88	0.54
≥2.6	0.82	0.71	0.62	0.87	0.53
≥2.7	0.82	0.74	0.65	0.87	0.55
≥2.8	0.80	0.76	0.66	0.87	0.56
≥2.9	0.78	0.78	0.68	0.86	0.57
≥3.0	0.75	0.79	0.68	0.84	0.54

LCQ, Leicester Cough Questionnaire; PGIC, patient global impression of change; ROC, receiver operating characteristic.
